# Circulating Monocytes Act as a Common Trigger for the Calcification Paradox of Osteoporosis and Carotid Atherosclerosis *via* TGFB1-SP1 and TNFSF10-NFKB1 Axis

**DOI:** 10.3389/fendo.2022.944751

**Published:** 2022-07-22

**Authors:** Ziliang Zeng, Rui Guo, Zheyu Wang, Haolin Yan, Xin Lv, Qiancheng Zhao, Xu Jiang, Chi Zhang, Di Zhang, Canchun Yang, Wenpeng Li, Zhilei Zhang, Qiwei Wang, Renyuan Huang, Bo Li, Xumin Hu, Liangbin Gao

**Affiliations:** Department of Orthopedics, Sun Yat-sen Memorial Hospital, Guangzhou, China

**Keywords:** CD14+ monocyte, calcification paradox, osteoporosis, carotid atherosclerosis, single cell sequence

## Abstract

**Background:**

Osteoporosis often occurs with carotid atherosclerosis and causes contradictory calcification across tissue in the same patient, which is called the “calcification paradox”. Circulating monocytes may be responsible for this unbalanced ectopic calcification. Here, we aimed to show how CD14^+^ monocytes contribute to the pathophysiology of coexisting postmenopausal osteoporosis and carotid atherosclerosis.

**Methods:**

We comprehensively analyzed osteoporosis data from the mRNA array dataset GSE56814 and the scRNA-seq dataset GSM4423510. Carotid atherosclerosis data were obtained from the GSE23746 mRNA dataset and GSM4705591 scRNA-seq dataset. First, osteoblast and vascular SMC lineages were annotated based on their functional expression using gene set enrichment analysis and *AUCell* scoring. Next, *pseudotime* analysis was applied to draw their differentiated trajectory and identify the key gene expression changes in crossroads. Then, ligand–receptor interactions between CD14^+^ monocytes and osteoblast and vascular smooth muscle cell (SMC) lineages were annotated with *iTALK*. Finally, we selected calcification paradox-related expression in circulating monocytes with *LASSO* analysis.

**Results:**

First, we found a large proportion of delayed premature osteoblasts in osteoporosis and osteogenic SMCs in atherosclerosis. Second, CD14^+^ monocytes interacted with the intermediate cells of the premature osteoblast and osteogenic SMC lineage by delivering TGFB1 and TNFSF10. This interaction served as a trigger activating the transcription factors (TF) SP1 and NFKB1 to upregulate the inflammatory response and cell senescence and led to a retarded premature state in the osteoblast lineage and osteogenic transition in the SMC lineage. Then, 76.49% of common monocyte markers were upregulated in the circulating monocytes between the two diseases, which were related to chemotaxis and inflammatory responses. Finally, we identified 7 calcification paradox-related genes on circulating monocytes, which were upregulated in aging cells and downregulated in DNA repair cells, indicating that the aging monocytes contributed to the development of the two diseases.

**Conclusions:**

Our work provides a perspective for understanding the triggering roles of CD14^+^ monocytes in the development of the calcification paradox in osteoporosis- and atherosclerosis-related cells based on combined scRNA and mRNA data. This study provided us with an elucidation of the mechanisms underlying the calcification paradox and could help in developing preventive and therapeutic strategies.

## Introduction

With the aging of the population, osteoporosis and carotid atherosclerosis have become common degenerative problems affecting people’s health and undermining quality of life (1, 2). Clinical observations show that osteoporosis often occurs with carotid atherosclerosis in the same patient, especially in postmenopausal women, causing reduced osteogenic bone mass and excessive calcification in blood vessels, which is called the “calcification paradox” (3). Recent studies have shown that the cytologic basis of the development of the calcification paradox of osteoporosis and atherosclerosis is generally unbalanced calcification across tissues, in which osteogenesis of the bone marrow osteoblast lineage is suppressed when the vascular intima suffers from excessive calcification due to an osteogenic phenotype of vascular smooth muscle cells (SMCs) (4, 5).

In recent decades, substantial evidence has shown that the calcification paradox involves multiple biological factors in the bone-vascular axis, including hormones, cytokines, ectoenzymes, inflammatory factors and exosomes (6, 7). A chronic inflammatory state contributes to the pathogenesis of both osteoporosis and atherosclerosis, in which circulating monocytes may act as a common trigger and cause unbalanced ectopic calcification (8–10). It remains unclear whether monocytes serve as a common trigger disturbing the cell state transition in the osteoblast and vascular SMC lineages, and the mechanism underlying unbalanced cross-tissue calcification has not yet been reported. In light of recent advancements, single-cell sequencing technology has enabled a comprehensive understanding of the roles of monocytes within environments related to the calcification paradox, namely, the bone marrow and blood vessels, at single-cell resolution (11, 12). Here, we aimed to elucidate the role of monocytes in the dysregulated differentiation of cells of the osteoblast lineage in osteoporosis and vascular SMC lineage in vascular calcification based on a combination of single-cell data and mRNA data.

**Figure 1:**
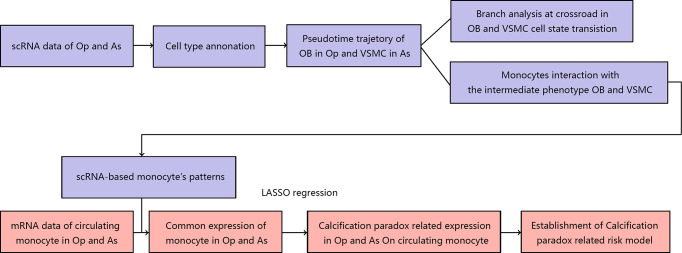
Design of the experiment and workflow of this study.

In this study, we integrated the scRNA and mRNA data of postmenopausal patients with osteoporosis and carotid atherosclerosis. First, we elucidated the landscape of the bone environment in osteoporosis and the vascular environment in atherosclerosis based on scRNA data. Then, we undertook a more thorough analysis of the osteoblastic lineage in the osteoporotic environment and the vascular SMC lineage in the atherosclerotic environment. On the one hand, we revealed the *pseudotime trajectory* of cell state transitions for the osteoblast and vascular SMC lineages. We also explored the internal and external factors driving the cell state transition at the *pseudotime trajectory* crossroads. Next, we explored the underlying cellular interaction between the osteoblast and vascular SMC lineages in the calcification paradox environment, especially the role of CD14^+^ monocytes. Finally, we compared the scRNA markers of monocytes with the mRNA expression patterns of circulating monocytes in osteoporosis and carotid atherosclerosis. We identified calcification paradox-related markers and established a calcification paradox-related risk scoring model based on the circulating monocyte expression profile. The workflow used in our study is summarized in **Figure 1**.

## Material and Methods

### Single-Cell Sequencing Data Sources

The single-cell data in our study were obtained from the *Gene Expression Omnibus (GEO) database*, and the data sources are summarized in **Table 1**. We obtained osteoporosis scRNA transcriptome sequencing data from *GSM4423510* (https://ftp.ncbi.nlm.nih.gov/geo/samples/GSM4423nnn/GSM4423510/) (12, 13), which was derived from a bone marrow biopsy from a 67-year-old postmenopausal osteoporotic patient. CD271^+^ bone marrow-derived mononuclear cells (BM-MNCs) were extracted from the tissue and sequenced on an *Illumina NovaSeq 6000* system. Carotid atherosclerosis scRNA transcriptome sequencing data were obtained from *GSM4705591 (* https://ftp.ncbi.nlm.nih.gov/geo/samples/GSM4705nnn/GSM4705591/ *)* (11, 14), which was derived from the carotid artery obtained from an endarterectomy of a 76-year-old postmenopausal patient with carotid atherosclerosis. Single cells were digested from atherosclerotic plaques of carotid arteries and sequenced on an *Illumina NovaSeq 6000* system.

**Table 1:** Data sources.

Project	Datasets	Sample size
**Single cell analysis using scRNA-seq**		
Osteoporosis data	*GSM4423510*	1
Patients source: 67-year-old postmenopausal osteoporotic patient; Tissue: CD271+ bone marrow derived mononuclear cells; Platform: *Illumina NovaSeq 6000 model*		
Carotid atherosclerosis data	*GSM4705591*	1
Patients source: 76-year-old postmenopausal patient with carotid atherosclerosis; Tissue: Carotid artery from endarterectomy; Platform: *Illumina NovaSeq 6000 model*		
**Calcification paradox related risk model using mRNA data**		
Osteoporosis data	*GSE56815*	40
Patients source: Postmenopausal patients with low and normal bone mass density; Tissue: Circulating monocytes from blood, isolated with *Monocyte-negative isolation kit*; Platform: *Affymetrix Human Genome U133A Array platform*		
Carotid atherosclerosis data	*GSE23746*	95
Patients source: Patients with and without carotid atherosclerotic plaque; Tissue: Circulating monocytes from blood, isolated with *Monocyte-negative isolation kit*; Platform: *Sentrix HumanRef-8 Expression BeadChip platform*		

### scRNA-seq Data Processing and Cell Annotation

The osteoporosis and carotid atherosclerosis scRNA-seq data were processed with the *Seurat* package in two separate pipelines (15). First, low-quality cells were excluded based on the types of genes detected, total number of detected genes, and percentage of mitochondrial genes. Second, the eligible data were normalized with the *LogNormalize* method, and 5000 hypervariable gene features were selected using the *variance-stabilizing transformation (VST)* method. Then, the single-cell expression profiles were scaled, and batch effects were removed. Next, principal component analysis (PCA) with 50 presumptive principal components (PCs) was applied, and a curve of the cumulative percent of variation was generated to obtain the final number of PCs. Finally, the profile was subjected to *uniform manifold approximation and projection (UMAP)* analysis and dimension reduction with a resolution of 1.0. We obtained markers of different clusters using the *FindAllMarkers* function with an adjusted *p* value (with the *Benjamin & Hochberg* method) threshold of 0.05 and *log fold change (logFC)* threshold of 0.25. Cell types of different clusters were annotated based on known cell markers.

### Differentiation Trajectory Analysis of Osteoblast and Vascular SMC Lineages

The differentiation trajectories of the osteoblast lineage in osteoporosis and the vascular SMC lineage in carotid atherosclerosis were evaluated with gene expression patterns and *pseudotime* analysis. For the gene expression pattern analysis, we used the *AUCell* score to assess the expressed gene sets of osteoblast lineage and vascular SMC lineage to infer the cell state and the transition order (16). Based on the inferred cell state, we used the *Monocle2* package to calculate cell state transition and *pseudotime* orders among different clusters (17). First, we filtered out genes used for clustering cells along the *pseudotime trajectory*. We computed the *mean-variance relationship* for each expressed gene among different cell clusters, and the following gene fitting criteria were included in the candidate list: (1) *average expression* ≥ 0.5 and (2) *dispersion empirical* ≥ 1*dispersion fit. The *differentialGeneTest* function was utilized to select the genes significantly altered along *pseudotime* order, with a *q* value threshold of 0.0001. Then, we performed dimension reduction using the *discriminative dimensionality reduction with trees* (*DDRTree*) method and ordered cells along the *pseudotime trajectory*. Next, we further investigated abnormal branch points for the osteoblast lineage and vascular SMC lineage. Generally, an abnormal branch point refers to a branch point of delayed osteoblastic differentiation in the osteoblast lineage and a branch point of osteogenic differentiation in the vascular SMC lineage. Branch-dependent expression was identified *via branched expression analysis modeling* (*BEAM*) with a *q* value threshold of 0.0001. Finally, we focused on the bifurcations of gene expression along the branches. Gene set enrichment analysis and transcriptional regulator analysis were performed with *Metascape* analysis (18).

### Ligand–Receptor Interactions Between Osteoblast Lineage and Vascular SMC Lineage and the Calcification Paradox Environment

The abnormal differentiation of osteoblast lineage and vascular SMC lineage is closely associated with the calcification paradox environment. We performed *ligand–receptor (LR) interaction* analysis to investigate the interaction between the osteoblast lineage and vascular SMC lineage in the calcification paradox environment using the *iTALK* package (19). Expressed genes were selected for LR interaction analysis according to the following criteria: (1) the top 20 highly expressed genes and (2) marker genes in corresponding clusters. Then, we selected the LR interactions that were biologically relevant to the calcification environment.

### Identification of Calcification Paradox-Related Markers in Circulating Monocytes and Construction of a Calcification Paradox-Related Risk Scoring Model Based on Circulating Monocytes

The mRNA data in this section are summarized in **Table 1**. We obtained osteoporosis mRNA transcriptome array data from *GSE56815* (https://ftp.ncbi.nlm.nih.gov/geo/series/GSE56nnn/GSE56815) (20, 21). These data were derived from circulating monocytes isolated with a *Monocyte-Negative Isolation Kit (Miltenyi Biotec, Inc.)* from 20 postmenopausal patients with low bone mass and 20 postmenopausal patients with normal bone mass. The mRNA data were sequenced on the *Affymetrix Human Genome U133A Array platform*. Carotid atherosclerosis transcriptome sequencing data were obtained from *GSE23746* (https://ftp.ncbi.nlm.nih.gov/geo/series/GSE23nnn/GSE23746) (22). These data were derived from circulating monocytes, which were also isolated with a Monocyte-Negative Isolation Kit, from 19 patients without carotid atherosclerosis and 76 patients with carotid atherosclerosis. The expression profiles were generated on the *Sentrix HumanRef-8 Expression BeadChip* platform. Since the mRNA data of circulating monocytes were generated on different platforms, we adjusted and removed batch effects between the two expression profiles based on *the empirical Bayes framework* using the *sva* package (23).

First, we obtained scRNA-based monocyte markers by intersecting the gene markers of monocyte clusters in osteoporosis and atherosclerosis data. Second, scRNA-based monocyte markers were used in univariate analysis of mRNA data from circulating monocytes in the context of osteoporosis and atherosclerosis to identify the common expression patterns between circulating monocytes and local monocytes in osteoporosis and atherosclerosis. Then, we identified calcification paradox-related markers among the commonly expressed genes of circulating monocytes based on the *least absolute shrinkage and selection operator (LASSO)* using the *glmnet* package (24). *LASSO* analysis was repeated 1000 times to achieve a satisfactory fit to the circulating monocyte features of osteoporosis and atherosclerosis. Finally, these predictors were utilized to develop a *binary logistic regression* model for scoring calcification paradox-related risk in osteoporosis and atherosclerosis based on circulating monocytes. The calcification paradox-related risk score was calculated with the following formula: $Y=\sum_{i=1}^{n}coef_{i}*X_{i}$ , where *“coef_i_”* and *“X”* denote the coefficient and expression level of each predictor. Ultimately, a *receiver operating characteristic (ROC)* curve was utilized to examine the performance of the predictive model, and an *area under the curve (AUC)* value of >0.70 indicated good performance.

## Result

### scRNA-seq Data Quality Control and Annotation

**Figure 2:**
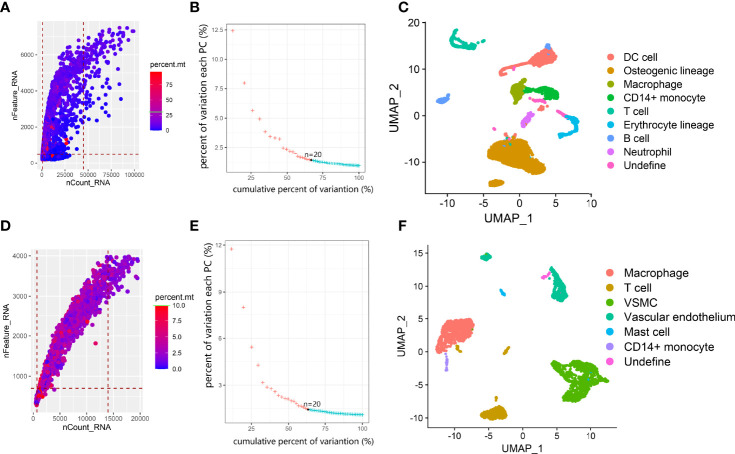
Overview of data processing and cell annotation in scRNA data. **(A, D)** Dot plot of data quality control in scRNA data. **(B, E)** elbow plot showing the percent of variation and cumulative percentages associated with each PC in scRNA data. **(C, F).** UMAP plot showing clusters of main cell types from osteoporosis and atherosclerosis scRNA-seq datasets.

The 10x Genomics scRNA-seq data of postmenopausal osteoporotic bone marrow (GSM4423510) and atherosclerotic carotid tissue (GSM4705591) were used in our study (13, 14). For the osteoporosis scRNA data, we found 5014 eligible single cells with data fitting the following criteria: (1) types of genes detected > 800; (2) total RNA counts between 500 and 45000; and (3) percentage of mitochondrial gene expression <30% (**Figure 2A**). First, the expression profile was normalized and scaled. Then, we set 20 dimensions in PCA and UMAP analysis, which yielded a 67.1% cumulative percentage of variation and 0.03% change in variation between neighboring PCs (**Figure 2B**). The cells were classified into clusters based on the UMAP algorithm (**Figure 2C**). Based on marker genes (listed in **Table 2**, the cluster annotation showed in **Supplementary Table 1**), the clusters were annotated as osteoblast lineage, dendritic cells (DCs), macrophages, CD14+ monocytes, erythrocytes, T cells and B cells. For the carotid atherosclerosis scRNA data, we identified 2767 eligible single cells with data fitting criteria: (1) types of genes detected > 700; (2) total RNA counts between 700 and 14000; and (3) percentage of mitochondrial gene expression <10% (**Figure 2D**). First, the expression profile was normalized and scaled. Then, we set 20 dimensions in PCA and UMAP analysis, which yielded a 63.4% cumulative percentage of variation and 0.03% change in variation between neighboring PCs (**Figure 2E**). The cells were classified into clusters based on the UMAP algorithm (**Figure 2F** **),** the cluster annotation showed in **Supplementary Table 2**). The clusters were annotated as vascular SMC lineage, vascular endothelium, macrophages, monocytes, mast cells and T cells.

**Table 2:** The canonical markers for the cell types.

Cell cluster	Marker genes
Osteoblastic lineage cells	RUNX2, ALPL, IBSP, SOST
CD14^+^ Monocytes	CCR2, CD14, CSF3R, FCGR1A, SELL, S100A8, S100A9, S100A12
Macrophage	C1, CD9, CD63, CD163, CSF1R, MRC1
Dendritic cells	TCF4, CXCR3, CD74, IL3RA
B cells	CD19, CD79
T cells	CD3, CD4, CD7, CD8
Neutrophils	CD11B, CD177
Erythrocyte lineage cells	ALAS2, EPB42, GATA1, KLF1, AHSP
Mast cells	CD63, RHOH, BTK, KIT, LAT, LAT2
Vascular smooth muscle cells	ACTA2, CNN1, CNN3, MYH, VIM
Vascular endothelium	CD34, VWF
Fibroblasts	DCN, FN1, COL1A1, COL1A2

### Differentiated Trajectory of Osteoblast Lineage in Osteoporosis

**Figure 3:**
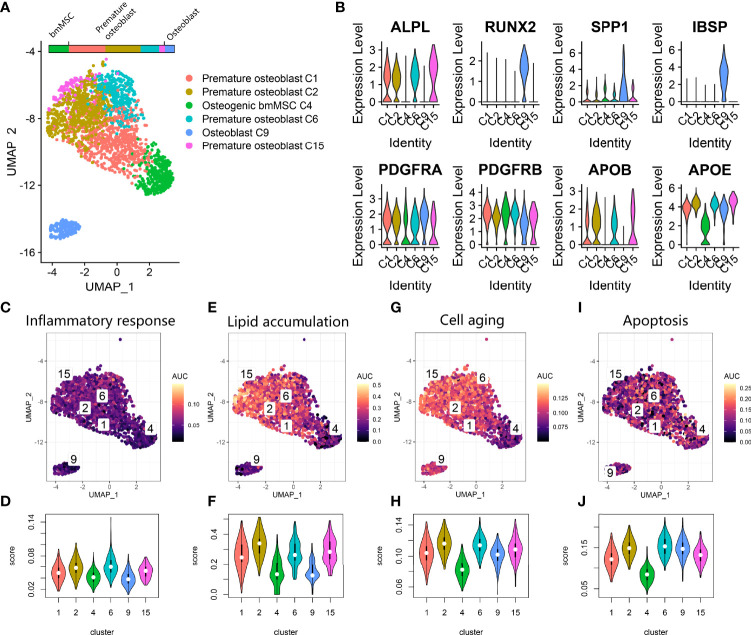
Osteoblast lineage expression in osteoporosis. **(A)** UMAP plot showing osteoblast lineage clusters. **(B)** Violin plot for the osteogenic gene (ALPL, RUNX2, SPP1, IBSP) and fat cell differentiation gene (PDGFRA, PDGFRB, APOB, APOE) expression markers in osteoblast lineage clusters. **(C, E, G, I).** Scatter plot showing the distribution of *AUCell* scores of gene sets, including inflammatory response **(C)**, lipid accumulation **(E)**, cell aging **(G)** and apoptosis **(I)**, in the osteoblast lineage. Yellow indicates a high score, and purple indicates a low score. **(D, F, H, J)** Violin plots exhibiting the *AUCell* scores of gene sets including inflammatory response **(D)**, lipid accumulation **(F)**, cell aging **(H)** and apoptosis **(J)** in the osteoblast lineage.

We further investigated osteoblast lineage clusters in the osteoporosis data **(** **Figure 3A** **).** First, based on osteogenic markers, we further annotated C4 as primitive osteogenic bmMSCs, which expressed low levels of osteogenic markers; C1, C2, C6, and C15 as premature osteoblasts, which expressed ALPL and low levels of SPP1 and IBSP; and C9 as mature osteoblasts, which expressed RUNX2, SPP1 and IBSP (**Figure 3B**) (25). To our surprise, premature osteoblasts accounted for the vast majority (76.3%) of osteogenic bmMSCs, and the transitional cells between premature osteoblasts and mature osteoblasts were absent on the UMAP plot. Additionally, we noticed that the osteoblast lineage highly expressed PDGFRA/PDGFRB, which is a hallmark of adipocyte differentiation, and apolipoprotein APOB and APOD, which participate in local lipoprotein particle accumulation (**Figure 3B**) (26). Using *AUCell* (**Figures 3C–J**), we found that inflammatory response expression was increased in C2 and C6. The lipoprotein particle gene set was significantly upregulated in the premature osteoblast clusters C1, C2, C6 and C15. The premature osteoblast clusters C1, C2, C6 and C15 also showed upregulated expression of genes involved in cell senescence and apoptosis compared with that of the primitive osteogenic bmMSC cluster C4.

**Figure 4:**
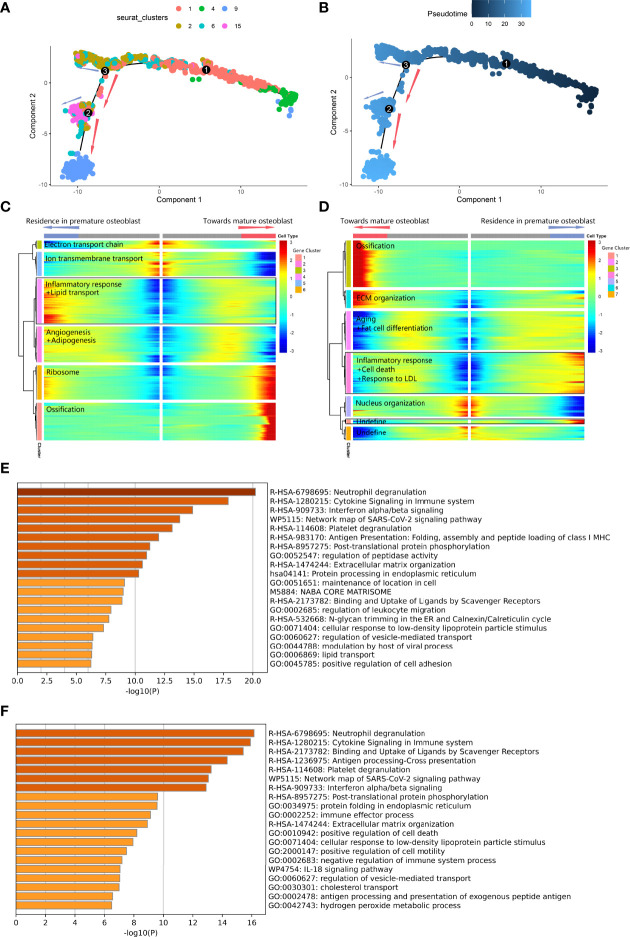
Differentiated trajectory of osteoblast lineage cells. **(A, B)** Scatter plot showing the *pseudotime trajectory* in the osteoblast lineage. **(A)** is colored with cell clusters, and **(B)** is colored with *pseudotime* orders. The blue arrow indicates delayed transition in premature osteoblasts. The red arrow indicates the transition toward mature osteoblasts. **(C, D).** Heatmap plot showing gene expression alterations along the branch at node 3 **(C)** and node 2 **(D)** on the *pseudotime trajectory* tree. Red indicates high expression, and blue indicates low expression. The black box indicates the highly expressed gene cluster in the premature osteoblast branch. **(E)** Bar plot for the top-ranked gene set enrichment in the highly expressed gene cluster in the premature osteoblast branch at node 3. **(F)** Bar plot for the top-ranked gene set enrichment in the highly expressed gene cluster in the premature osteoblast branch at node 2.

Second, we performed *pseudotime* analysis to comprehensively explore the abnormal gap between premature osteoblasts and mature osteoblasts along the differentiation trajectory. The cell-state transition from primitive osteogenic bmMSCs (C4) to premature osteoblast clusters (C1, C2, C6 and C15) was consecutive, while the transition from premature osteoblast clusters to mature osteoblasts (C9) was discontinuous along the *pseudotime trajectory* (**Figures 4A, B** **)**. The differentiation trajectory was interrupted at branch nodes 2 and 3 (*BEAM* result in **Supplementary Table 3**). At node 3 (**Figures 4C–E** **)**, we found an increase in ossification and ribosomal protein expression along the branches toward node 2, while the inflammatory response and lipid transport were increased along another branch. At downstream node 2 (**Figures 4D–F** **)**, we found a further increase in ossification-related gene expression and ECM development expression along the branches toward the transition to C9, while the expression of factors related to the inflammatory response and cell death was upregulated along another branch. Overall, after combining nodes 2 and 3, we noticed that high expression of factors related to lipid transport, inflammatory response and cell death could lead to delayed cell state transition from premature osteoblast clusters to mature osteoblast clusters.

### Differentiated Trajectory of Vascular SMC Lineage in Carotid Atherosclerosis

**Figure 5:**
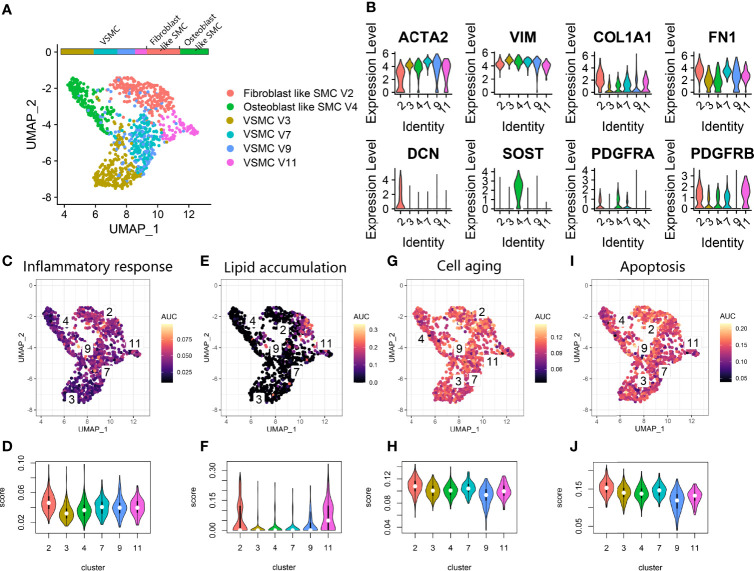
Vascular SMC lineage expression in atherosclerosis. **(A)** UMAP plot showing vascular SMC lineage clusters. **(B)** Violin plot for the smooth muscle cell marker genes (ACTA2, VIM), fibroblast marker genes (COL1A1, FN1, DCN), osteoblast marker genes (SOST) and fat cell differentiation genes (PDGFRA, PDGFRB) expression markers in vascular SMC lineage clusters. **(C, E, G, I).** Scatter plot showing the distribution of *AUCell* scores of gene sets, including inflammatory response **(C)**, lipid accumulation **(E)**, cell aging **(G)** and apoptosis **(I)**, in the vascular SMC lineage. Yellow indicates a high score, and purple indicates a low score. **(D, F, H, J)** Violin plots exhibiting the *AUCell* scores of gene sets, including inflammatory response **(D)**, lipid accumulation **(F)**, cell aging **(H)** and apoptosis **(J)**, in the vascular SMC lineage.

We also investigated the vascular SMC lineage in the vascular calcification data **(** **Figure 5A** **)**. Clusters V3, V4, V7, V9, and V11 expressing ACTA2 and VIM were annotated as vascular SMC phenotypes. We noticed that V2 was characterized by high COL1A1, FN1, and DCN expression and annotated as fibroblast-like SMCs (**Figure 5B**). V4 was characterized by high SOST expression and annotated as an osteoblast-like SMC (**Figure 5B**). Fibroblasts such as SMCs and osteoblasts such as SMCs commonly participate in osteoid formation in atherosclerotic plaques and are classified as osteogenic SMCs. Additionally, some V2, V4, and V7 coexpressed the adipocyte differentiation markers PDGFRA and PDGFRB. By using *AUCell* scoring (**Figures 5C–J** **)**, we found that V2 showed upregulation of factors related to the inflammatory response, cell aging and apoptosis, while V9 showed downregulation of factors related to cell aging and apoptosis among the vascular SMC clusters. V2 and V11 showed activation of lipoprotein particle formation. Overall, among various vascular SMC clusters, we identified V2 and V4 as osteogenic SMCs and V3, V7, V9 and V11 as vascular SMC-derived intermediate cells (11).

**Figure 6:**
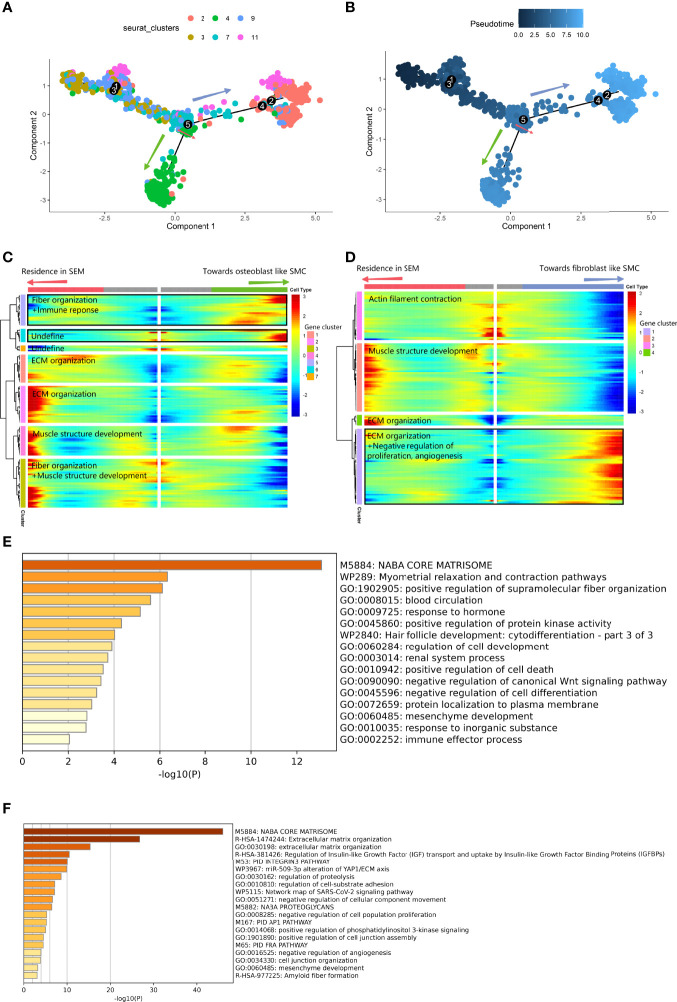
Differentiated trajectory of vascular SMC lineage cells. **(A, B)** Scatter plot showing the *pseudotime trajectory* in the vascular SMC lineage. **(A)** is colored with cell clusters, and **(B)** is colored with *pseudotime* orders. The green arrow indicates the transition toward osteoblast-like SMCs. The blue arrow indicates the transition toward fibroblast-like SMCs. **(C, D).** Heatmap plot showing gene expression alterations along the branch at node 5 toward osteoblast-like SMCs **(C)** and toward fibroblast-like SMCs **(D)** on the *pseudotime trajectory* tree. Red indicates high expression, and blue indicates low expression. The black box shows the highly expressed gene cluster in osteoblast-like and fibroblast-like SMCs. **(E)** Bar plot for the top-ranked gene set enrichment in the high expression gene cluster in the osteoblast-like SMC branch at node 5. **(F)** Bar plot for the top-ranked gene set enrichment in the high expression gene cluster in the fibroblast-like SMC branch at node 5.

Second, we applied *pseudotime* analysis to explore the cell state transitions among different vascular SMC phenotypes. The results showed a cell-state transition from vascular SMCs to osteogenic SMCs (V2, V4) along the *pseudotime trajectory* (**Figures 6A, B** **)**. The differentiation trajectory was interrupted at branch node 5. At node 5, the vascular SMC-derived intermediate cells were at the intersection of maintaining the typical phenotype or converting to the osteogenic phenotypes(*BEAM* result in **Supplementary Table 4**). On the branch toward osteoblast-like cells (V4) (**Figures 6C–E** **)**, expression related to positive regulation of cell death, immune response and negative regulation of cell differentiation was increased. On the branch toward fibroblast-like cells (V2) (**Figures 6D–F** **)**, activation of ECM organization and suppression of cell proliferation and angiogenesis were observed.

### LR Interaction Analysis of Osteoblast and Vascular SMC Lineages at the Bifurcation Node of the Differentiation Trajectory Tree

**Figure 7:**
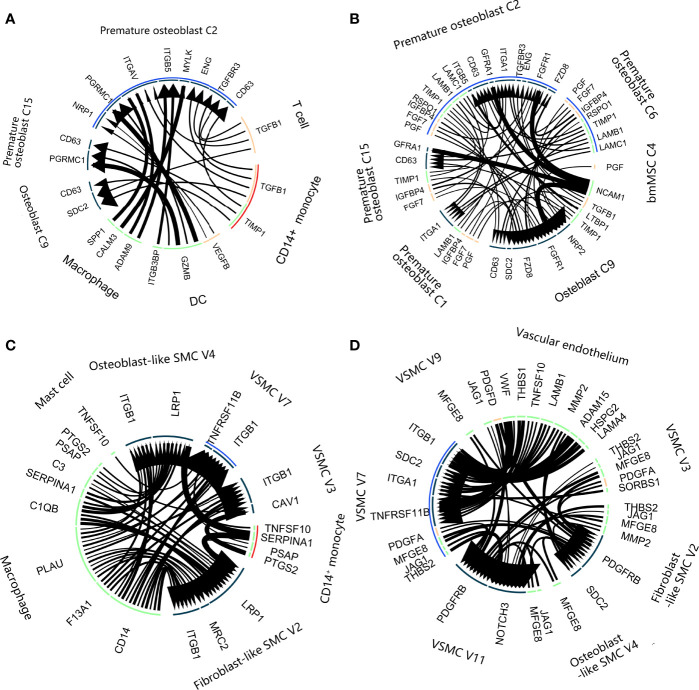
Ligand–receptor interactions in calcification paradox-related bone and vascular environments. **(A, B).** Ligand–receptor interaction plot in the bone environment with osteoporosis. **(A)** Interaction between the osteoblast lineage and other cell types in the environment. **(B)** Interaction among the osteoblast lineages. The arrow direction indicates the direction of the ligand–receptor interaction, in which the arrow is the receptor and the nock is the ligand. The thickness of the line indicates the relative expression levels from high to low. The blue arc covered intermediate states C2 and C6. The red arc covers the C14^+^ monocytes. **(C, D).** Ligand–receptor interaction plot in the vascular environment with atherosclerosis. **(A)** Interaction between the vascular SMC lineage and other cell types in the environment. **(B)** Interaction among the vascular SMC lineage. The arrow direction indicates the direction of the ligand–receptor interaction, in which the arrow is the receptor and the nock is the ligand. The thickness of the line indicates the relative expression levels from high to low. The blue arc covered the intermediate state V7. The red arc covers the C14^+^ monocytes.

We performed LR interaction analysis to explore the influence of the LR interaction on the cell state transition at the bifurcation node of the differentiation trajectory tree. Regarding the osteoporosis data **(** **Figure 7A**, LR interaction listed in **Supplementary Table 5** **)**, bifurcated node 2 consisted of mostly premature osteoblasts (C2 and C6). C2 accounted for most LR interactions among the osteoblast lineage with the bone environment. These interactions could be divided into 3 categories. The first group involved the sensing of various growth factor stimuli, including TGFB1 from CD14^+^ monocytes and T cells and VEGFB from DCs. The second group involved macrophage secretion of SPP1 and CALM3, which promoted differentiation toward mature osteoblasts. The third group involved DCs, which also interacted with C2 *via* GZMB : PGRMC1, which could lead to cell death. We also developed a paracrine LR network for the osteoblast lineage **(** **Figure 7B** **)**, including FGF7, IGFBP4, PGF, TGFB1, SPP1 and TIMP1. Mature cells in C9 delivered LTBP1, controlling the release of local TGFB1 to cells in C2 and C6.

Regarding the vascular calcification data, trifurcated node 5 contained mostly intermediated vascular SMC phenotypes (V7). The LR interaction between V7 and the vascular environment generally consisted of an inflammatory interaction with macrophages, including C1QB, C3, and CD14 (**Figure 7C**, LR interaction listed in **Supplementary Table 6**). We noticed that V7 interacted with CD14^+^ monocytes and mast cells *via* TNFSF10:TNFRSF11B, which is known to inactivate its inhibition of NFκB signaling pathways (27). V7 had a close relationship with other vascular SMC phenotypes and the vascular endothelium, which mainly consisted of 2 aspects (**Figure 7D**). On the one hand, they were stimulated with VCAN, VWF, CTGF, PDGF and JAG1:NOTCH3 to accelerate angiogenesis. On the other hand, they achieved cell adhesion to vascular cells through ITGA1, ITGB1, FBN1, and FN1. Additionally, they secreted LTBP1 and LTBP3 to delay and control the absorption of local TGFB1 by V7.

### Gene Expression Alterations in Osteoblast Lineage With Delayed Osteoblastic Maturation and Vascular SMC Lineage Undergoing Transition to Osteogenic Phenotypes

**Figure 8:**
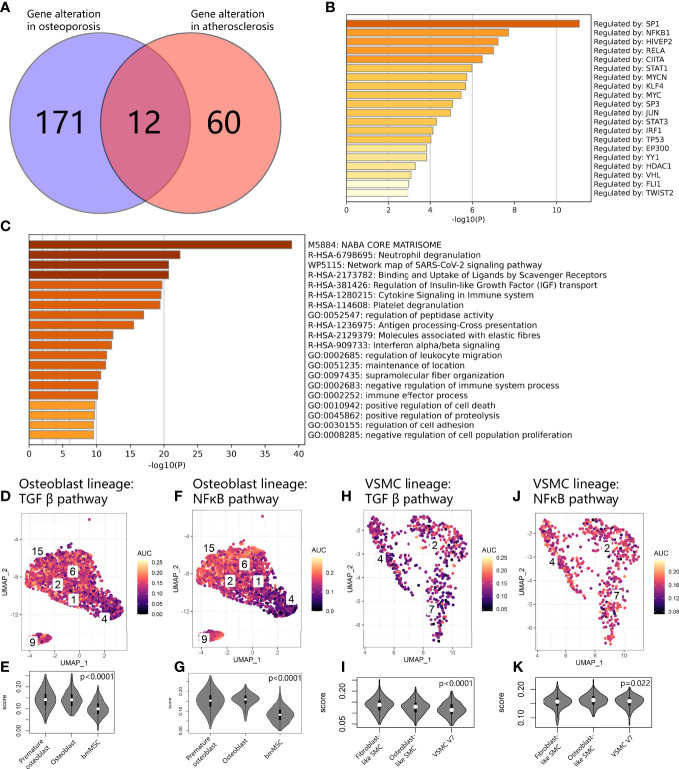
Common expression patterns at the crossroads of osteoblast and vascular SMC cell state transitions. **(A)** Venn diagram showing gene alterations between delayed exit from the premature state in the osteoblast lineage and osteogenic and fibrogenic transition in vascular SMCs. **(B)** Bar plot for the top-ranked transcriptome factors on alteration expression at the crossroads of the osteoblast and vascular SMC cell state transitions. **(C)** Bar plot for the top-ranked gene set enrichment in altered expression at the crossroads of the osteoblast and vascular SMC cell state transitions. **(D, F, H, J)** Scatter plot showing the distribution of *AUCell* scores of gene sets, including the TGFβ pathway **(D–H)** and NFκB **(F–J)**, in osteoblast and vascular SMC lineages. Yellow indicates a high score, and purple indicates a low score. **(E, G, I, K).** Violin plots exhibiting the *AUCell* scores of gene sets, including the g TGFβ pathway **(E–I)** and NFκB **(G–K)**, in osteoblast and vascular SMC lineages.

We compared the gene expression alterations between bmMSCs with delayed osteoblastic maturation in osteoporosis and vascular SMCs undergoing the transition to fibroblastic and osteogenic phenotypes in vascular calcification and obtained the union gene expression alterations shared by these two processes in the calcification paradox. As a result, we identified 243 consistently upregulated genes (**Figure 8A**). These genes were enriched in immune response and expression related to positive regulation of cell death and negative regulation of proliferation (**Figure 8C**). The TRRUST results suggested that the alterations were regulated by SP1 and NFKB1 (**Figure 8B**). Then, we explored the KEGG database and found that the activity of the transcription factor SP1 was activated by phosphorylation through TGFβ signaling pathways and that NFKB1 could be activated by NFκB signaling pathways. This result supported the LR interaction finding that TGFB1 and the TNFSF10:TNFRSF11B interaction play key roles in the development of the calcification paradox. Additionally, we found that the TGFβ signaling pathways and NFκB signaling pathway *via* IKK were relatively upregulated in both premature osteoblast clusters and intermediate phenotypes of vascular SMCs (V7) (**Figures 8D–K**).

### Gene Expression Patterns of CD14+ Monocytes in the Calcification Paradox

**Figure 9:**
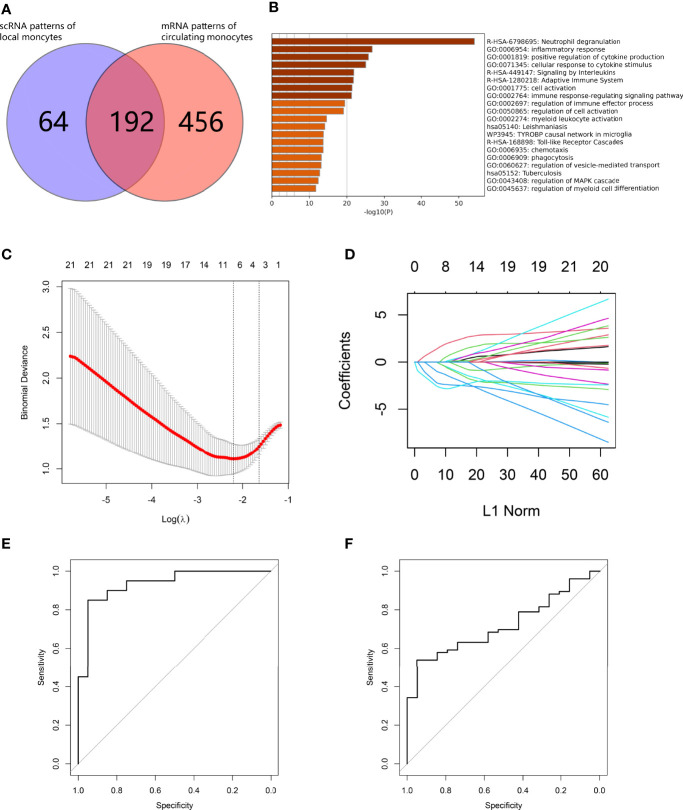
Development of a calcification paradox risk scoring model. **(A)** Venn diagram showing common genes between scRNA-based markers of CD14^+^ monocytes and mRNA-based markers of circulating CD14^+^ monocytes in both diseases. **(B)** Bar plot for the top-ranked gene set enrichment in common genes between scRNA-based markers of CD14^+^ monocytes and mRNA-based markers of circulating CD14^+^ monocytes in both diseases. **(C–F).** establishment of the Calcification Paradox Risk Scoring Model on circulating CD14^+^ monocytes in both diseases. **(C)** Calculation paradox expression features on circulating CD14^+^ monocyte selection in the *LASSO* model. **(D)** Coefficient curves of the calcification-related gene curves. **(E)** ROC curves to assess the accuracy of the Calcification Paradox Risk Scoring Model on circulating CD14^+^ monocytes to predict osteoporosis in the training groups. **(F)** ROC curves to assess the accuracy of the Calcification Paradox Risk Scoring Model on circulating CD14^+^ monocytes to predict atherosclerosis in the validation groups.

From LR interaction analysis, we identified CD14^+^ monocytes as one of the triggers leading to abnormal cell state transition in both osteoblast and vascular SMC lineages. We further investigated calcification paradox-related markers in osteoporotic and atherosclerotic circulating monocyte mRNA datasets. First, based on their markers, the CD14^+^ monocyte cluster was annotated as CD14^+^ CD16^-^ G1 classic monocytes. For fitting to scRNA-based monocyte phenotypes, we selected circulating monocyte mRNA series from postmenopausal osteoporotic patients (GSE56815) and from carotid atherosclerosis patients (GSE23746), both of which were isolated by negative selection with CD3, CD7, CD16, CD19, CD56, CD123 and CD235a (*Miltenyi - Monocyte Isolation Kit II*) (21, 22). Second, since the two mRNA series were sequenced on different array platforms, we adjusted the expression profile to remove batch effects between arrays. Third, we identified 249 common gene markers commonly upregulated in both bone and vessel environments associated with the calcification paradox. The calcification paradox-related markers intersected with the upregulated genes in circulating monocytes in both osteoporosis and atherosclerosis patients, with a total of 192 genes (**Figure 9A**, listed in **Supplementary Table 7**). We noticed that most (76.49%) scRNA-based monocyte calcification paradox-related markers were upregulated in mRNA from circulating monocytes in osteoporosis and atherosclerosis patients. Using enrichment analysis, these genes could be divided into 3 groups (**Figure 9B**). The first group was related to chemotaxis and the inflammatory response. The second group was related to detoxification of reactive oxygen species (ROS) and cell impairment due to ROS stress. The third group was related to the cell response to lipids and was associated with atherosclerosis. Next, we repeated iterations of the *LASSO* algorithm and selected the model that showed good performance in both osteoporosis and atherosclerosis. On the one hand, we used osteoporosis data as the training dataset and atherosclerosis data as the validation group. In the model, we selected 7 calcification paradox-related markers and established the formula for the *Calcification Paradox Risk Score* as follows: *Y* = 61.599 – 2.787 * *H*3*F*3*A* – 2.369 * *ARPC*3 – 1.251 * *PAK*1 – 0.383 * *DOCK*2 – 0.366 * *BAZ*1*A* + 1.811 * *MAP*3*K*1 + 0.374 * *PABPC*1 (**Figures 9C, D** **),** score listed in **Supplementary Table 8**). *The Calcification Paradox Risk Score* was reliable and robust for predicting osteoporosis and atherosclerosis based on *ROC curves* (*AUC_osteoporosis_* = 0.94; *AUC_atherosclerosis_* = 0.72) (**Figures 9E, F** **)**. A higher calcification paradox risk score was related to an increased risk of calcification paradox (calcification paradox v control: 40.58 (39.92-41.37) v 39.19 (38.64-40.05), p<0.001). We noticed that 2 genes were positively correlated and 5 genes were negatively correlated with calcification paradox risk (**Table 3**). Among the negatively correlated genes, MAP3K1 and PABPC1 were associated with cellular aging. Among the negatively correlated genes, H3F3A, ARPC3, PAK1 and BAZ1A participated in DNA repair in the response to DNA damage.

**Table 3:** Calcification paradox-related genes based on the *LASSO* algorithm.

Gene name	Protein name	Correlation with risk
ARPC3	Actin-related protein 2/3 complex subunit 3	Negative
BAZ1A	Bromodomain adjacent to zinc finger domain protein 1A	Negative
DOCK2	Cell growth regulator with EF hand domain protein 1	Negative
H3F3A	Histone H3.3	Negative
MAP3K1	Mitogen-activated protein kinase kinase kinase 1	Positive
PABPC1	Polyadenylate-binding protein 1	Positive
PAK1	Serine/threonine-protein kinase PAK 1	Negative

## Discussion

In clinical observations, aging patients, especially postmenopausal patients, often suffer from reduced osteogenesis and bone mass accompanied by abnormal mineral deposition in blood vessels, and this contradictory situation is called the calcification paradox (3). In recent decades, substantial evidence has highlighted the close relationship between osteoporosis and carotid atherosclerosis. The bone-vascular axis has been implicated *via* multiple biological factors, including inflammatory factors, oxidative stress compounds, hormones, and circulating cells and factors, which lead to abnormal osteoblast and vascular SMC cell state transitions in lesions (6). Monocytes play a critical role in the regulation of osteogenesis and inflammation in both osteoporosis and vascular calcification, but the understanding of their roles in the postmenopausal bone-vascular axis in the context of the calcification paradox remains rudimentary (5, 8). In light of recent advancements, scRNA-seq has enabled us to reveal monocyte cellular interactions with calcification paradox-related bone marrow and vessel environments at single-cell resolution (11, 12). In this study, we applied single-cell analysis to postmenopausal osteoporosis and carotid atherosclerosis data to explore the role of monocytes in disturbing osteoblast and vascular SMC cell state transitions and identified calcification paradox-related markers of circulating monocytes in the context of the calcification paradox.

Delayed maturation of osteoblasts and osteogenic SMCs are known to be indispensable cytological bases for the development of osteoporosis and atherosclerosis (4, 5). Our study revealed the single-cell landscape and showed that the majority (76.3%) of osteoblastic lineages were blocked in the premature osteoblast stage, when osteogenic SMCs accounted for nearly half of vascular SMCs (41.9%). The population was consistent with a previous scRNA-based report of osteoporosis and atherosclerosis, supporting our cell annotation and the critical roles of these cells in both diseases (11, 12). Delayed premature osteoblasts (C2, C6) showed impaired osteogenic expression, and osteogenic SMCs (V2, V4) showed high expression of SOST and COL1A1, FN1 and DCN, leading to osteoid deposition at the vascular intima. We noticed that delayed premature osteoblasts and osteogenic SMCs showed activation of cell senescence and apoptosis. Cell senescence is one of the internal factors leading to abnormal cell state transition in the osteoblast lineage in osteoporosis and in the vascular SMC lineage in atherosclerosis (28, 29). This finding supported the idea that the accumulation of premature osteoblasts and osteogenic SMCs accounted for the altered osteoid organization in osteoporosis and atherosclerosis.

There has been substantial evidence that the reprogramming of the cell state transition in cells of the osteoblast lineage and vascular SMC lineage is triggered by both internal factors from their altered expression and external factors from the bone and vascular environment (11, 12). By using *pseudotime* analysis, we found that osteoblast lineage maturation was mainly blocked at C2 and C6 in the osteoporotic environment, while osteogenic SMCs (V2, V4) were derived from the intermediate state SMC V7. Regarding internal factors, both processes showed upregulation of inflammatory response-related expression along the trajectory branches. This finding was consistent with the understanding that a chronic inflammatory state could lead to aging and disturbed reprogramming in osteoblast and vascular SMC differentiation (30, 31). Based on *TRRUST* analysis, altered expression during two processes was regulated by the transcription factors (TF) SP1 and NFKB1, both of which were also reported as regulators in modulating bone and vascular remodeling in an inflammatory environment (32, 33). Regarding external factors, CD14^+^ monocytes closely interacted with intermediate states C2 and V7 by delivering TGFB1 and TNFSF10 to activate the TGFβ and NFκB signaling pathways. This was consistent with the internal alterations, in which premature osteoblast C2 and C6 and intermediate state SMC V7 were relatively upregulated in the TGFβ and NFκB signaling pathways. Such features have been demonstrated to be involved in the development of osteoporosis and atherosclerosis (34–36). Through comprehensive scRNA-seq analysis, we showed that CD14^+^ monocytes could act as triggers in the TGFβ and NFκB signaling pathways in the osteoblast and vascular SMC lineages, which led to their downstream activation of the transcription factors SP1 and NFKB1 to push the intermediate cells toward delayed premature osteoblastic phenotypes and osteogenic SMC phenotypes.

Overall, our study revealed that CD14+ monocytes contributed to the development of both osteoporosis and carotid atherosclerosis. Recent studies highlighted the critical roles of monocyte infiltration in both diseases; circulating CD14+ monocytes could migrate from the vessels and undergo chemotaxis to bone marrow and vascular intima lesions (37). We noticed that most (76.49%) common scRNA-based CD14+ monocyte markers between osteoporosis and atherosclerosis were upregulated in the CD14+ circulating monocytes from osteoporosis and atherosclerosis patients. The commonly differentially expressed genes were enriched in factors related to chemotaxis and inflammatory responses. This finding suggested that circulating CD14+ monocytes were similar to local CD14+ monocytes in postmenopausal osteoporotic bone marrow and atherosclerotic vascular intima lesions, suggesting that circulating monocytes could be a common trigger in the calcification paradox. Additionally, it also indicated that active monocytes also expressed CD52, CYBA and CYBB, which were associated with excessive local ROS production in tissues, causing the senescence-associated secretory phenotype (SASP) and revealing a possible mechanistic link between osteoporosis and carotid atherosclerosis (38, 39). Then, we mined the calcification paradox-related genes among the commonly expressed genes. *LASSO* analysis is accepted as one of the most common bioinformatic methods to select markers among multiple candidates, accompanied by risk models to examine its stability (40). Therefore, we identified 7 genes related to the calcification paradox through a repeated fitting model, which showed stable performance in assessing the risk of osteoporosis and atherosclerosis (*AUC_osteoporosis_* = 0.92; *AUC_atherosclerosis_* = 0.72). Among them, 2 genes (MAP3K1 and PABPC1) were positively correlated, and 5 genes (ARPC3, BAZ1A, DOCK2, H3F3A and PAK1) were negatively correlated with the calcification paradox risk. On the one hand, overexpression of MAP3K1 and PABPC1 could induce cellular senescence and was reported in several aging-related diseases (41, 42). On the other hand, the downregulated H3F3A, ARPC3, PAK1 and BAZ1A were related to DNA repair in response to DNA damage. Interestingly, previous clinical observations showed that concomitant administration of statins and vitamin D in postmenopausal patients could benefit osteoporotic progression, while antiresorptive drugs failed to provide satisfactory antiatherosclerotic performance (43–45). The administration of statins and vitamin D helped to maintain the antioxidative, anti-inflammatory and membrane stabilizing environment, which partly supported our finding since it benefited in reducing the aging monocyte-induced impairment when simply targeting the bone environment could hardly stop the vicious cycle between the two diseases. This finding suggests that the administration of antioxidative and anti-inflammatory treatments could act as a potential treatment in the cooccurrence of postmenopausal osteoporosis and atherosclerosis. In summary, our findings showed that aging and impaired circulating monocytes were chemotactic to bone and vessel lesions and contributed to promoting the development of the calcification paradox.

There were some limitations of our study. First, our study was performed by combining and comparing the gene expression patterns of postmenopausal osteoporosis and carotid atherosclerosis patients. The experimental verification of calcification paradox-related markers in circulating monocytes from postmenopausal patients with coexisting osteoporosis and atherosclerosis is necessary to clarify the association between circulating monocytes and the development of coexisting osteoporosis and atherosclerosis. Second, due to the absence of circulating monocyte mRNA data from patients with postmenopausal carotid atherosclerosis, we used a data series from male carotid atherosclerosis patients, which would account for the decreased performance in the validation with atherosclerosis. Third, our study did not consider early intervention in high calcification paradox risk patients. Further improvements of the model could focus on exploring the latent clinical manifestations of and interventions for patients at high risk of the calcification paradox.

## Conclusion

In this study, we provided a perspective for understanding the roles of CD14^+^ monocytes in the development of the calcification paradox in osteoporosis- and atherosclerosis-related cells based on combined scRNA and RNA data. First, using scRNA data, we found a large proportion of delayed premature osteoblasts in osteoporosis and osteogenic SMCs in atherosclerosis. Second, CD14^+^ monocytes interacted with the intermediate cells, which were at the crossroads of the osteoblast and vascular SMC cell state transitions, by delivering TGFB1 and TNFSF10. This interaction served as the trigger activating the transcription factors SP1 and NFKB1 to upregulate the inflammatory response and cell senescence and led to a delayed premature state in the osteoblast lineage and osteogenic transition in vascular SMCs. Then, using bulk RNA data, we found that most expression patterns in monocytes from osteoporosis and atherosclerosis patients were present in circulating monocytes from both diseases. Circulating monocytes are characterized by the activation of factors associated with chemotaxis, the inflammatory response and the cell response to stress, such as ROS and lipid stimuli. Finally, we identified 7 circulating monocyte calcification paradox-related genes based on the expression patterns of circulating monocytes. The calcification paradox-related markers suggested that circulating monocytes undergo cell aging and activation of DNA repair in response to DNA damage.

## Data Availability Statement

The original contributions presented in the study are included in the article/**Supplementary Material**. Further inquiries can be directed to the corresponding authors.

## Ethics Statement

The studies involving human participants were reviewed and approved by Institute Ethics committee in the Sun Yat-sen Memorial Hospital. Written informed consent for participation was not required for this study in accordance with the national legislation and the institutional requirements.

## Author Contributions

GL, LB and HX conceived the studies. ZiZ, GR, WZ, YH designed the research process and were major contributors in writing the manuscript. ZQ and JX collected and assembled the data in the GEO databases. ZC, ZD, YC, LX, ZhZ, WQ, and HR participated in software support and data analysis. All authors read and approved the final manuscript.

## Funding

The study was supported by the Science and Technology Program of Guangzhou, China (201707010089), Medical Science and Technology Research Foundation of Guangdong Province, Guangzhou, China (A2021371), Funding of Basics and Application Basics of Guangzhou (202102020096), and Funding of Regenerative Medicine and Health Laboratory of Guangdong (1102101201).

## Conflict of Interest

The authors declare that the research was conducted in the absence of any commercial or financial relationships that could be construed as a potential conflict of interest.

## Publisher’s Note

All claims expressed in this article are solely those of the authors and do not necessarily represent those of their affiliated organizations, or those of the publisher, the editors and the reviewers. Any product that may be evaluated in this article, or claim that may be made by its manufacturer, is not guaranteed or endorsed by the publisher.
